# An integrative approach to investigate the respective roles of single-nucleotide variants and copy-number variants in Attention-Deficit/Hyperactivity Disorder

**DOI:** 10.1038/srep22851

**Published:** 2016-03-07

**Authors:** Leandro de Araújo Lima, Ana Cecília Feio-dos-Santos, Sintia Iole Belangero, Ary Gadelha, Rodrigo Affonseca Bressan, Giovanni Abrahão Salum, Pedro Mario Pan, Tais Silveira Moriyama, Ana Soledade Graeff-Martins, Ana Carina Tamanaha, Pedro Alvarenga, Fernanda Valle Krieger, Bacy Fleitlich-Bilyk, Andrea Parolin Jackowski, Elisa Brietzke, João Ricardo Sato, Guilherme Vanoni Polanczyk, Jair de Jesus Mari, Gisele Gus Manfro, Maria Conceição do Rosário, Eurípedes Constantino Miguel, Renato David Puga, Ana Carolina Tahira, Viviane Neri Souza, Thais Chile, Gisele Rodrigues Gouveia, Sérgio Nery Simões, Xiao Chang, Renata Pellegrino, Lifeng Tian, Joseph T. Glessner, Ronaldo Fumio Hashimoto, Luis Augusto Rohde, Patrick M.A. Sleiman, Hakon Hakonarson, Helena Brentani

**Affiliations:** 1 Inter-institutional Grad Program on Bioinformatics, University of São Paulo, São Paulo, SP, Brazil; 2 Center for Applied Genomics, The Children’s Hospital of Philadelphia, Philadelphia, PA, USA; 3 Department & Institute of Psychiatry, University of São Paulo Medical School, São Paulo, SP, Brazil; 4 National Institute of Developmental Psychiatry for Children and Adolescents (INCT-CNPq), São Paulo, SP, Brazil; 5 Department of Psychiatry, Hospital de Clínicas de Porto Alegre, Federal University of Rio Grande do Sul, Porto Alegre, RS, Brazil; 6 Department of Psychiatry, Federal University of São Paulo, São Paulo, SP, Brazil; 7 Center of Mathematics, Computation and Cognition. Universidade Federal do ABC, Santo André, Brazil; 8 Hospital Israelita Albert Einstein, Clinical Research, São Paulo, SP, Brazil; 9 Federal Institute of Espírito Santo, Serra, ES, Brazil; 10 Mathematics & Statistics Institute, University of São Paulo, São Paulo, SP, Brazil; 11 Department of Pediatrics, The Perelman School of Medicine, University of Pennsylvania Philadelphia, PA, USA

## Abstract

Many studies have attempted to investigate the genetic susceptibility of Attention-Deficit/Hyperactivity Disorder (ADHD), but without much success. The present study aimed to analyze both single-nucleotide and copy-number variants contributing to the genetic architecture of ADHD. We generated exome data from 30 Brazilian trios with sporadic ADHD. We also analyzed a Brazilian sample of 503 children/adolescent controls from a High Risk Cohort Study for the Development of Childhood Psychiatric Disorders, and also previously published results of five CNV studies and one GWAS meta-analysis of ADHD involving children/adolescents. The results from the Brazilian trios showed that cases with *de novo* SNVs tend not to have *de novo* CNVs and vice-versa. Although the sample size is small, we could also see that various comorbidities are more frequent in cases with only inherited variants. Moreover, using only genes expressed in brain, we constructed two “in silico” protein-protein interaction networks, one with genes from any analysis, and other with genes with hits in two analyses. Topological and functional analyses of genes in this network uncovered genes related to synapse, cell adhesion, glutamatergic and serotoninergic pathways, both confirming findings of previous studies and capturing new genes and genetic variants in these pathways.

In the last decade, multiple studies have investigated the genetic susceptibility for ADHD, most notably including assessment of enrichment of copy number variation^1^,^2^,^3^,^4^,^5^,^6^,^7^. Genome-wide association studies (GWAS) of single nucleotide variants (SNVs) in ADHD have also been executed and a few variants of small or moderate effect sizes have been reported^3^,^8^,^9^. Despite these promising results, genes identified by GWAS (capturing CNVs and SNVs) still only contribute for a small percentage of the heritability of this complex trait^10^ and replication across these studies has been inconsistent^11^. This is likely attributed to the combination of modest effect sizes of causative mutations on disease susceptibility and underpowered studies due to small sample sizes. Meta-analyses in ADHD, while theoretically more powerful, has not succeeded in discovering novel loci^7^. However, copy-number variation (CNVs) analysis has been more successful than SNV analysis^1^,^2^,^4^,^5^,^12^,^13^,^14^,^15^.

Extensive literature has also emerged from the integration of different databases to prioritize variants of biological interest involving complex diseases such as psychiatric disorders^16^,^17^,^18^,^19^,^20^. In this regards, the public database ADHDgene (http://adhd.psych.ac.cn/) and reported a candidate ADHD gene list based on the overlap of five prioritization gene tools. Poelmans *et al*.^19^ used the results from five previous GWAS in ADHD to search for biological functions and pathways with the objective of reanalyze the results together. They performed a systematic literature searches for (putative) functions of the proteins coded by 85 consensus genes from GWAS.

Protein-protein interaction networks have more recently been shown to be of added value in: identifying new disease genes, studying their network properties, identifying disease-related sub-networks, and network-based disease classification^21^. The foundation for using data integration based on protein-protein interactions (PPI) data relies on the hypothesis that proteins related to the same diseases or phenotypes tend to interact or are closely located in the network^22^,^23^. Approaches using GWAS data and PPI networks have been published with focus on psychiatric disorders, including bipolar disorder^16^, schizophrenia^17^, autism^18^, as well as ADHD^1^,^14^. Elia and colleagues^1^ used PPI data to investigate networks with genes overrepresented in CNVs affecting multiple cohorts. They reported that CNVs in glutamate receptor family genes and in genes interacting with them were enriched in approximately 10% of cases, showing for the first time association of glutamate receptor genes with ADHD.

With the development of new genomic techniques to investigate changes in DNA, it has been possible to discover the genetic causes for a larger range of conditions and phenotypes than ever before. For some complex disorders, it has been necessary to apply methods to analyze genome-wide variants with SNP arrays in conjunction with sequencing data to combine both common and rare variants^24^,^25^,^26^. Analysis of high throughput sequencing data has been successful in covering genomic regions with low frequency mutations, which also have impact in complex diseases^14^,^25^. Moreover different types of very rare variants, such as *de novo* variants, compound heterozygosity and even heritable heterozygous missense variations have been shown to contribute to neurodevelopment disorders^27^,^28^.

In other complex psychiatric disorders, such as autism^29^,^30^,^31^ and schizophrenia^32^,^33^, the role of common or inherited variants has been shown to be important. Small CNVs have also been reported^34^,^35^. These studies show that the genetic landscape of neurodevelopment psychiatric disorders can be composed by the combination of different types of variants.

According to Hawi *et al*.^36^, “As with other psychiatric disorders, the monogenic concept of ADHD has now been supplanted by a more plausible polygenic hypothesis where multiple risk genes (each of minor/modest effect) contribute to the etiology of the disorder. As detailed previously, ADHD-associated genes (and those showing trends towards association) are scattered through the genome but tend to be enriched within specific functional categories. This suggests that the emphasis on any individual candidate gene should be shifted to consider a broader network view of biological pathways involving ADHD-implicated genes.”

As previously proposed by Girirajan and Eichler^37^, we have used in this present study both *de novo* and inherited variants to integrate our model of ADHD genetic architecture using an exome analysis of 30 sporadic ADHD Brazilian trios and SNP-array from 503 typically Brazilian developed children. To validate our findings we integrated our results with public ADHD genetic resources, gene expression data bases and PPI databases exploring the ADHD network in accordance to Hawi *et al*.^36^ and the hypothesis of network medicine described by Barabasi *et al*.^22^, that genes related to the same disease tend to interact.

## Methods

In all analyses we only accepted variations in genes expressed in brain based on data from the Brain Span Atlas of the Developing Human Brain (http://brainspan.org), Brain Atlas (http://human.brain-map.org) and GTEx Portal (http://gtexportal.org). The analyses were conducted in 3 steps:

1. we used only data from Brazilian samples, searching for *de novo* and inherited SNVs and CNVs comparing ADHD trios and control samples to better explore the genomic architecture of our ADHD samples.
2. we constructed a protein-protein interaction network (PPI) based on all genes with SNVs and CNVs findings by our work and studies with samples of children and adolescents reported by ADHDgene database (http://adhd.psych.ac.cn) and explore functional annotation of the network as well as topological properties of ADHD genes in the PPI network.
3. to achieve a highly reproducible ADHD gene set we selected only genes with two evidences: presented in our SNV and CNV analyses in different families or presented in our analyses and also in a previous GWAS or CNV public ADHD analyses. We used this gene set as seeds to growth a PPI.

**Figure 1:**
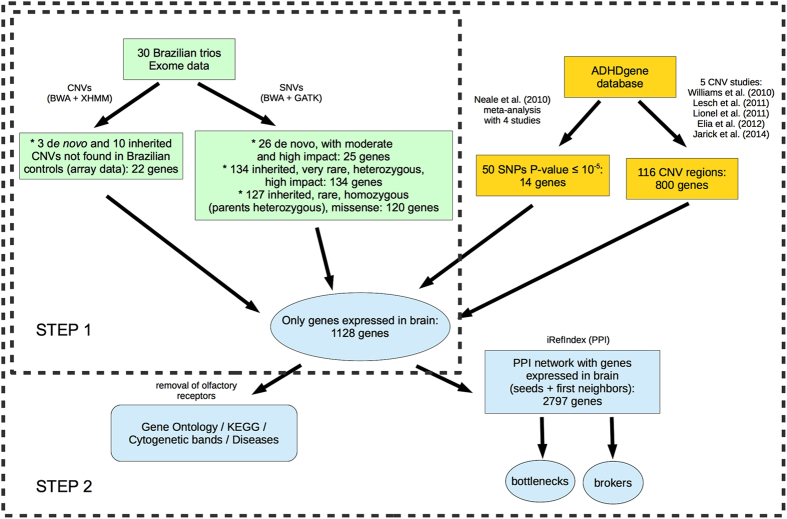
Summary of two first steps of the analyses performed in this work. The boxes in the topleft, in green, show the data the came from the exome analysis of Brazilian trios. The boxes in the top right, in yellow, show the data the came from previously reported findings in public databases. In the center and in the bottom, the blue elements show what was done with the combination of genes in both sets of data.

A summary of steps 1 and 2 can be found in Fig. 1.

### Brain expression data

The samples from Brain Span Atlas of the Developing Human Brain (http://brainspan.org), Allen Human Brain Atlas (http://human.brain-map.org) and GTEx Portal (http://www.gtexportal.org) were used to understand what genes are expressed in brain. Genes with 1 *reads per kilobase per million reads* (RPKM) in any area of the brain in at least one sample were considered expressed. After evaluation, our list contained 54141 transcripts.

STEP 1 - Brazilian samples

The sample is part of the High Risk Cohort Study for Psychiatric Disorders in Childhood^38^, a two-stage large community study of children aged 6 to 12 years (at screening) from 57 schools in two Brazilian cities - Porto Alegre (n = 22) and São Paulo (n = 35). The ethics committee of the University of São Paulo approved the study and written consent from parents of all participants were collected. Detailed data could be found in Salum *et al*.^38^. Briefly, during the screening phase at their school registry day, 9,937 informants were interviewed. Two subgroups using a random-selection (n = 958) and high-risk group (n = 1,514) selection procedure that consisted of selecting individuals with high family loading of symptoms and ongoing psychiatric symptoms, were recruited resulting in a total sample of 2,512. Saliva was collected from all subjects and their parents. To establish child psychiatric diagnoses, the parent-reports of the Brazilian Portuguese version of the Development and Well-Being Assessment – DAWBA was performed by trained psychiatrists and to assess parent psychiatric diagnoses the Mini International Psychiatric Interview (MINI) and MINI Plus were used. Out of 2,512 subjects 240 met DAWBA criteria for any ADHD, being 66 from the randomly selected group and 240 from the high-risk group. For the exome analysis, we selected 35 trios, in which the child was affected by ADHD and the parents were healthy as well as no familial history of ADHD was reported in the screening interview. A sub-sample of 724 subjects out of the 2,512 was selected to collect blood and perform a functional magnetic resonance imaging (fMRI) study^39^. We used 503 subjects from this sub-sample, in which the child was not affected by any DAWBA diagnosis and has available genotypes after quality control criteria using an SNP-array platform assay.

### Exome data processing of Brazilian samples

The Nextera^®^ Exome Enrichment Kit (Illumina Inc., San Diego, USA) was utilized to construct the libraries, which were prepared over a target region of 62 Mb, 20,794 genes, 201,121 exons. The sequencing was performed using two technologies. In eight trios, Illumina HiScan SQ™ (Illumina, Inc.) was utilized. In 27 other trios, HiSeq 2500™ (Illumina, Inc.) was utilized. The reads were sequenced with the paired-end technique, producing in average 24 million reads per sample with length of 100bp, resulting in 2.4Gb sequenced per sample. The reads were mapped to *National Center for Biotechnology Information* (NCBI) *reference human genome build* 37 (GRCh37/hg19), using the software Burrows-Wheeler Aligner, or BWA (http://bio-bwa.sourceforge.net). These mapped reads were the input for Picard (http://broadinstitute.github.io/picard) and GATK Unified Genotyper (https://www.broadinstitute.org/gatk) to find single-nucleotide variants through the best practices pipeline (https://www.broadinstitute.org/gatk/guide/best-practices) and calculate the exons coverage to run the program to identify CNVs. We used the software KING (http://people.virginia.edu/~wc9c/KING) to check kinships between parents and children. It is expected that the value of the kinship between one parent and one child must be between 0.17 and 0.35. If the kinship is much lower than this value, it means that there is no relation of parenthood or that the samples might be contaminated. Trios with at least one kinship relation below 0.19 were removed from our analyses.

### Single-nucleotide variant (SNV) analysis in exome of Brazilian cases

Variants were annotated using Annovar (http://annovar.openbioinformatics.org) and SnpEff (http://snpeff.sourceforge.net), and databases dbSNP build 137 (http://www.ncbi.nlm.nih.gov/SNP/). We called ‘common’ the variants with maximum value minor allele frequency (MAF) greater than 5% in the ExAC database (http://exac.broadinstitute.org). Variants with at maximum MAF between 1% and 5% were called ‘rare’, ‘very rare’ if the maximum MAF was less than 1% and ‘novel’ if the variant was not found in the databases. After the annotation, we looked for three types of SNVs that could contribute to the disease: 1. *de novo* mutations with moderate and high impact (missense, nonsense or splice site); 2. rare, moderate impact (missense) homozygous mutations for loci where both parents are heterozygous; and 3. very rare, high impact (nonsense and splice site) heterozygous mutations.

### Copy-number variant (CNV) analysis in SNP array data of Brazilian healthy children

The samples were genotyped in HumanCore-12 v1.0 BeadChip (Illumina, Inc.), with 298,930 SNPs. We used PennCNV^40^ to detect the CNVs in Brazilian control samples.

### Copy-number variant (CNV) analysis in exome of Brazilian cases

We used XHMM (http://atgu.mgh.harvard.edu/xhmm) to find copy-number variants in exome sequencing data. We selected only CNVs with at least 3 exons, minimum of 1kb, phred-scaled qualities of some CNV event in the interval and the quality of the region not being diploid were at least 90 and that were not present in Brazilian controls (SNP arrays data). We considered false positive and removed from the analysis CNVs present in more than three healthy parents, deletions with heterozygous SNVs rate above 10%, and duplications with average B allele frequency^40^ between 0.4 and 0.6.

STEP 2 –Integration of Brazilian data with public databases and ADHD PPI network construction.

### Single-nucleotide polymorphisms in public data

We used the set of genes related to the 50 resulting SNPs with P-value ≤ 10^−5^ from Neale *et al*.^7^ meta-analysis. This meta-analysis included only studies with children/adolescents and with the same categorical diagnostic variables, which decreases chances of phenotypic heterogeneity. All genes expressed in brain inside the CNV regions were considered.

### Copy-number variants in public data

We also used the CNV studies from predominantly Caucasian samples of children/adolescents reported in the ADHDgene database. They are part of the following publications: Williams *et al*.^5^ (57 CNVs reported), Lesch *et al*.^2^ (17 CNVs reported), Lionel *et al*.^4^ (23 CNVs reported), Elia *et al*.^1^ (19 CNVs reported) and Jarick *et al*.^15^ (1 CNV reported). From the regions reported by these studies, we searched for genes that had an overlap with these regions, using package *biomaRt* (http://bioconductor.org/packages/biomaRt). We converted all the regions to hg19 (version 19 of the human genome reference) positions using the package *rtracklayer* (http://bioconductor.org/packages/rtracklayer) of R language, which uses the same algorithm of UCSC genome browser (http://genome.ucsc.edu/cgi-bin/hgLiftOver). All genes expressed in brain inside the CNV regions were considered.

### Protein-protein interaction data

To construct the PPI networks, we used the data from iRefIndex compilation (http://irefindex.org). This database is a union of the main PPI databases: BIND (http://bind.ca), BioGRID (http://thebiogrid.org), CORUM (http://mips.helmholtz-muenchen.de/genre/proj/corum), DIP (http://dip.doe-mbi.ucla.edu), HPRD (http://hprd.org), IntAct (http://www.ebi.ac.uk/intact), MINT (http://mint.bio.uniroma2.it/mint), MPPI (http://mips.helmholtz-muenchen.de/proj/ppi)and OPHID (http://ophid.utoronto.ca). We selected only links supported by at least two pieces of direct experimental evidence demonstrating physical interaction between each pair of human proteins. The network was treated as an undirected graph. This final network has 73911 edges, representing protein-protein interactions, and 12464 nodes, representing genes/proteins.

### Network measures

In order to analyze the nodes and topological features, centrality measures (brokering centrality and betweenness centrality) and clustering coefficients were calculated^41^. The degree is the number of links of a node with other nodes (called neighbors) in the network. Nodes with very high degree are called hubs since they are connected to many neighbors. The removal of such nodes has great impact on the network topology. It has been shown that biological networks tend to be robust against random perturbations, but disruption of hubs often leads to system failure. In this work, a measure called brokering centrality^41^ was used to identify hubs whose neighbors do not connect each other (or they connect less than expected). This measure uses the degree and the clustering coefficient to be calculated. The betweenness centrality is the measure of how a node is in the center of the network, and it shows important nodes that lie on a high proportion of paths between other nodes in the network. Proteins with high betweenness centrality have been termed “bottlenecks”, for their role as key connectors of proteins with essential functional and dynamic properties^22^. For a review of formulas of all these measures, see Cai *et al*.^41^. To compute nodes/genes measures, it was used the Python library NetworkX (http://networkx.github.io). We calculated such measures for all the nodes in the network and classified the top 5% ranked for each measure as, respectively, brokers or bottlenecks.

### Analysis of overrepresented pathways, biological functions, cytogenetic bands and diseases associated to the different sets of genes

To perform the enrichment analyses, the tool WebGestalt (http://bioinfo.vanderbilt.edu/webgestalt) was utilized. In this program, the set of genes in the human genome was used as reference, the corrections were made using Benjamini and Hochberg method, and only the top 10 terms with an adjusted P-value less than 0.05 were considered significant for our analysis. For KEGG analysis, we used the program STRING (http://string.embl.de), because the version was more updated (2014) than WebGestalt for KEGG (2011). For STRING, the Bonferroni correction was utilized in the adjustment.

STEP 3 – To achieve a highly reproducible ADHD gene set we selected only genes harboring probably deleterious variations presented by at least two evidences: presented in Brazilian SNV and CNV analyses of two different families or presented in our analyses and also in a previous public analyses. Using only directed interaction to growth the seed genes considered highly reproducible we use the iRefIndex database (http://irefindex.org), to construct a PPI network and explore functional ontology of the network as already described above.

## Results

Psychiatric disorders are considered polygenic and multifactorial, so today it is clear that both rare and common variation contribute to the genetic architecture of such diseases. In addition, it is also known that individuals from different families could have different variations for the same diseases^42^. For these reasons, stringent analysis criteria have been used, as comparison with public databases in the gene level, not in the variant level. On the other hand, various disorders hare the same genes, and one gene could participate in different processes, which leads us to believe that we should look at such diseases in a systemic view. One way to investigate it is by searching for biological networks and its topological properties, like clusters of genes and their biological functions, which could better explain genetic mechanisms of predisposition for a range of diseases or even for a specific disease. Following this idea, to prioritize genes and networks related to ADHD we present our results in three steps:

1. Analysis of individual variations in a specific population (SNV and CNV of Brazilian samples);
2. Integration of our data with public data in a gene level and analysis of such data in a systems biology approach, using protein-protein interaction networks of genes expressed in brain (Integrated PPI ADHD network);
3. Finding a highly reproducible ADHD gene set and the ADHD network.

To have a high reliability in each of these steps, the following criteria detailed in the method section were used:

In the first one we describe our exome results of 30 trios using high stringent criteria to variant call: target coverage of 45× (average) and 50% of target regions with at least 20× of coverage. We removed trios with bad overall mapping quality and low values of kinship. Regarding overall single-nucleotide variants (SNVs), we removed variants with less than 10 read depth or genes not expressed in brain. More specifically for *de novo* SNVs, we selected only moderate to high impact (nonsense, missense or splice site) SNVs. For copy-number variants (CNVs), we used an independent sample of healthy Brazilian children/adolescents and removed any CNVs also found in this sample. Besides applying the best practices of XHMM (a reliable software for CNV calling in exoma data)^43^, we developed a new method for removing false positive CNVs taking into account single-nucleotide variants (SNVs) for both deletions and duplications. Our method, inspired by PennCNV^40^ algorithm, performs a post-processing analysis checking the allele frequencies of SNVs in each CNV to remove those with a large difference of expected proportions.

In the second part we used data from public GWAS data exactly to find more confident results. Up to date, none of the previous GWAS studies performed in children and adolescents with ADHD was able to reach statistical significance in genomic level. Although no variant reached significance, some findings show that common and rare variants could contribute to the disease. Given that, we decided to integrate our exome data results and public datasets of main genetic studies – including 50 SNPs from Neale *et al*.^7^ meta-analysis with P-value ≤ 10^−5^, and four studies that reported validated CNVs^1^,^2^,^4^,^5^ – all performed with children/adolescents having ADHD. Genes with no expression in brain, according to GTEx database, were removed.

In the third step, to achieve a highly reproducible ADHD gene set we selected only genes harboring probably deleterious variations presented by at least two evidences: presented in Brazilian SNV and CNV analyses of two different families or presented in our analyses and also in a previous public analyses. Using only directed interaction to growth the seed genes considered highly reproducible we use the iRefIndex database (http://irefindex.org), to construct a PPI network and explore functional ontology of the network as already described above.

1. Analysis of individual variations in a specific population (SNV and CNV in Brazilian samples).

### Single-nucleotide variants (SNV) analysis in the exome of Brazilian samples

Five trios were excluded from the analysis: one because the father sample did not pass the quality criteria of the sequencing pipeline, 3 trios because at least one kinship factor was less than 0.17 and one trio because both kinship factors were exactly at 0.17. Thirty trios remained in the analysis. We looked for three types of single-nucleotide variants that can be contributing to the disease:

- *De novo* mutations with moderate and high impact (missense, nonsense or splice site): 26 variants were found in 25 genes, and all of them are expressed in brain (Table 1). In the gene *VWDE* two variants were found in the same family. One cytogenetic band presented hits in three different families: 19p13 (size: 20 Mb).
- Very rare, high impact (nonsense and splice site) heterozygous mutations (Sup. Table 2): 134 different variants were found in 134 genes expressed in brain. Six out of these 134 genes presented the same variant in two families*: ACOXL, ANKRD42, CYFIP2, MSR1, NPSR1, OBSL1.* The other 128 genes presented only one variant. Five cytogenetic bands presented hits in at least 4families: 3q13 (size: 89.7Mb), 5q33 (size: 10.1 Mb), 11p15 (size: 21.7 Mb), and 19p13 (size: 20 Mb); and 19q13 (size: 26.7 Mb) presented hits in 10 families.
- Rare, moderate impact (missense) homozygous mutations for loci where both parents are heterozygous: 127 different variants were found in 120 genes expressed in brain. Five genes *(ACSM1, GIMAP6, ILDR1, MUC6, RGS12)* presented two variants in the same family, one gene *(IFLTD1)* presented three variants in the same family and one gene presented *(DNAH3)* the same variant in two different families. The other 113 genes presented only one variant. Two cytogenetic bands presented hits in 4 different families: 1p36 (size: 28 Mb) and 19q13 (size: 26.7 Mb). The other results are detailed in Sup. Table 3.

### Copy-number variant (CNV) analysis in the exome of Brazilian samples

After mapping the reads to the Human genome (GRCh37/hg19), we used the XHMM software to call copy-number variations (CNVs). We found 13occurrences of putative CNVs in the Brazilian children, three being *de novo* CNVs, seven inherited exclusively from father and three inherited exclusively from mother. In these regions, 22 genes were found to be expressed in brain. The Sup. Table 1 has all CNVs found in the 30 trios. The gene TRIM48 (11q11) was found in a *de novo* and in an inherited CNV in two different children. We used the software BEDTools (http://bedtools.readthedocs.org) to find the overlap and calculate the distances between CNV regions.

### Analysis of SNVs and CNVs in the Brazilian trios

**Figure 2:**
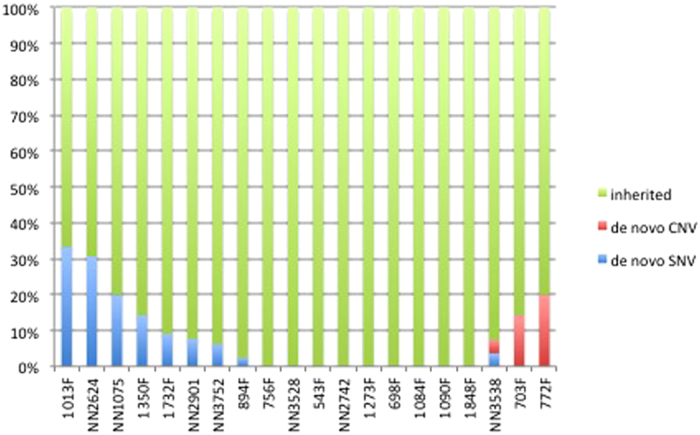
Barplot with percentages of inherited and *de novo* variants (divided in CNVs and SNVs) in Brazilian children found in our whole exome analysis.

To investigate how inherited and *de novo* variants were distributed in the Brazilian children, we merged inherited SNVs and CNVs and compared it with *de novo* SNVs and *de novo* CNVs (Fig. 2). It is interesting to note that although our sample size is small we could observe that in average, children with only inherited variants, when compared to children with *de novo* variants, have two times more comorbidities. This is shown with more details in Sup. Table 4.

2. Integration of our data with public data in a gene level and analysis of such data in a systems biology approach, using protein-protein interaction networks of genes expressed in brain (Integrated PPI ADHD network).

Taking into account all the genes reported with SNP or in CNV in public databases or genes with SNV or in CNV in the Brazilian data, we ended up with a set of 1128 genes expressed in brain. We removed the olfactory receptors (total = 80) to avoid bias in the functional analysis. The final set has 1048 genes.

The GO analysis of these genes showed significant results for molecular function “ligand-gated ion channel activity” (GO:0015276), with 18 genes and adjusted P-value of 0.0093. The KEGG analysis showed significant results for “Neuroactive ligand-receptor interaction” (22 genes, adj. P-value = 9.63e–05), “Metabolic pathways” (50 genes, adj. P-value = 0.0015), “Calcium signaling pathway” (14 genes, adj. P-value = 0.0025), “Tight junction” (12 genes, adj. P-value = 0.0025), “Ubiquitin mediated proteolysis” (12 genes, adj. P-value = 0.0025), “Adherens junction” (8 genes, adj. P-value = 0.0056), “Cytokine-cytokine receptor interaction” (15 genes, adj. P-value = 0.0238). The cytogenetic bands significantly associated with ADHD were 15q11 (88 genes, adj. P-value = 1.30e–87, size = 6.7 Mb), 16p11 (70 genes, adj. P-value = 6.82e–55, size = 8.5 Mb), 7p15 (20 genes, adj. P-value = 8.26e–12, size = 7.9 Mb), 11q25 (10 genes, adj. P-value = 3.86e–08, size = 4.2 Mb), 4p15 (15 genes, adj. P-value = 6.02e–08, size = 24.5 Mb), 15q12 (20 genes, adj. P-value = 2.69e–07, size = 2.4 Mb), 20p13 (15 genes, adj. P-value = 3.22e–07, size = 5.1 Mb) and 8q21 (16 genes, adj. P-value = 4.08e–07, size = 49.8 Mb).

**Figure 3:**
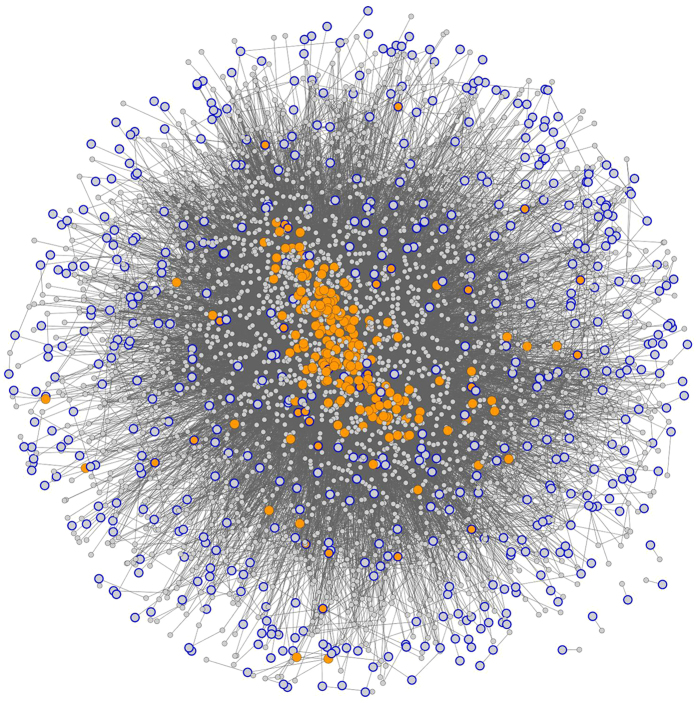
Network created with genes related with ADHD and the direct neighbor protein on PPI databases. The genes with blue borders are the seeds. The genes in orange are brokers or bottlenecks. Only 5.5% of the seeds are central (highly connected nodes).

To study how the genes were relatively located in the network space, we plotted a network with the genes related to ADHD and their direct interactions using protein-protein interaction databases. This network is composed only of genes expressed in brain. Out of 1128 genes, only 557 were mapped in the interactome. Besides the 557 seed genes, 2240 more genes were added to the network. The network is shown on Fig. 3.

3. Finding a highly reproducible ADHD gene set and the ADHD network.

To obtain a more robust view of the genes with SNVs and/or in CNVs, we selected genes with variants found in both the Brazilian exome data and public ADHD CNV data. Table 2 includes these 30 of the most significant and informative genes and which type of analyses they were derived from. All the other genes resulted from a single analysis.

**Figure 4:**
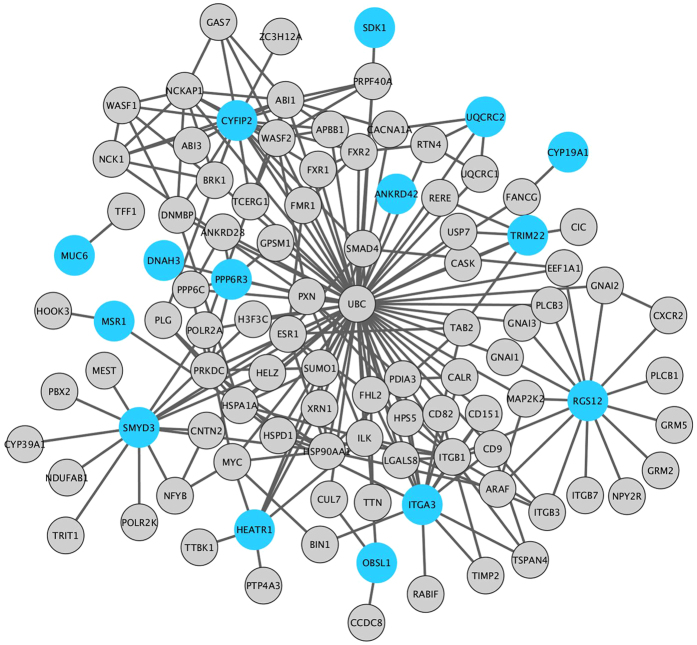
Network with genes in at least two analyses (seeds, which are the blue nodes) and their direct interactions (grey nodes).

Using only these 30 genes as seeds, we built a subnetwork adding the direct interactions to analyze which biological functions were associated with these genes. Out of 30 genes, only 15 were in the interactome. The final network ended up having 101 genes (Fig. 4). Using WebGestalt, we found gene ontology categories and protein modules over-represented in this network. Regarding cellular components, there are 13 genes related to neuron projection (adj. P-value = 6.00e–04), 5 genes related to cell-substrate adherens junction (adj. P-value = 3.00e–03), 34 genes related to membrane-enclosed lumen (adj. P-value = 1.60e–03) and 14 genes related to synapse (adj. P-value = 7.80e–05). Moreover, the central nervous system genes are significantly connected in the network, according to WebGestalt analysis of protein modules (adj. P-value = 3.02e–05). Using STRING, we performed the enrichment analysis for KEGG and obtained the more specific synapse results: glutamatergic synapse (7 genes, adj. P-value = 3.28e–04) and serotoninergic synapse (6 genes, adj. P-value = 4.81e–03). This is shown in Table 3.

## Discussion

Despite a 70% to 80% heritability estimate^44^, finding gene variants associated with the ADHD phenotype has been extremely challenging. Some genes seem to be more related to the susceptibility of any psychiatric neurodevelopmental disorder but the complexity of combinations of non-specific variations with more individual ones and the environment will bring the colors of a specific disorder^45^. With this idea, we explored different types of genetic variations using ADHD trios exome data. An intuitive method to find variations related to a disorder is to look for *de novo* variants in trios where the parents do not have ADHD and the children show the phenotype. Such variants have been shown as potential causes of several psychiatric diseases^12^,^13^,^34^,^46^. Here we found 26 *de novo* SNVs with moderate and high impact in 25 brain-expressed genes in 14 different families and three *de novo* CNVs in different families. Although combining exome and GWAS studies has some technical limitations, studies in the literature have demonstrated the effectiveness of using combined analysis methods^47^. To search CNVs related to ADHD, it is important to mention that we compared in this present study the copy-number variants found in the Brazilian trios using whole-exome data combined with CNV regions found in 503 Brazilian controls using SNP-array data. The intention was to remove any CNVs that are present in controls from the analysis.

However, it also has been shown that rare inherited variants^35^ play an important role in other neurodevelopment disorders^37^. Assuming that ADHD should not be different from other neurodevelopmental disorders, we searched for inherited SNVs and CNVs. Regarding SNVs, we found 127 rare, moderate impact (missense) homozygous mutations for loci where both parents are heterozygous in 120 reported genes expressed in brain, suggesting a possible recessive heritability in ADHD as has been suggested for autism^48^. Besides that, 134 very rare, high impact (nonsense and splice site) heterozygous mutations were found in 134 genes expressed in brain. Regarding structural variants, we found 10inherited CNVs that were not present in Brazilian controls. Although we found putative CNVs inherited more from father than from mother, the limitation of our small sample size makes difficult to assert that this is a recurring pattern.

Regarding the variants found only in the Brazilian cases, it was observed that usually it was necessary the combination of exclusively inherited variants or inherited variants with a few *de novo* variants. Interestingly, the children who presented *de novo* SNVs did not present *de novo* CNVs, and vice-versa (only one child is an exception, with one *de novo* CNV and one *de novo* SNV). This corroborates what has been shown in other psychiatric diseases^37^, such as autism^29^,^30^,^31^, schizophrenia^32^,^33^, and bipolar disorder^49^. In these diseases, the studies reported the role of *de novo* and inherited variants, as well as common and rare variants. Particularly in ADHD, Yang and colleagues^14^ performed a case-control analysis using SNP-array approach (Affymetrix 6.0) of a large cohort of Chinese subjects aiming to discover both common and rare variants associated with ADHD. Although they did not find genome-wide significant results, they could point some biological pathways using network analysis integrating the genes with higher association. More recently, Martin and colleagues^50^ used UK-based children samples to test whether children with ADHD with large rare CNVs had different polygenic risk scores (based on SNPs) for ADHD when compared to children with ADHD without these CNVs. In other words, their results implied that comparing children with a large, rare CNV with children without, the former require less of a polygenic burden to develop the disease, when we look at the SNPs in these children. However, our exome data made possible to look at smaller CNVs, and we also found CNVs with less than 100kb that could be playing a role in the disease.

When we combined the variants found in CNVs or SNVs taking into account the findings in our exome data and in the public databases of children with ADHD, the results highlighted interesting biological functions and diseases significantly associated to ADHD. For example, the GO molecular function and KEGG exhibited terms related to activity of cells in brain, as “ligand-gated ion channel activity”, of which some genes have been studied as therapeutic targets for ADHD, Alzheimer’s disease, schizophrenia, depression and tobacco addiction. Other pathways found have already been related to ADHD. The most significant were “Neuroactive ligand-receptor interaction” (the ADHDgene database reports 89 genes), “Calcium signaling pathway” (66 genes on ADHDgene) and “Cytokine-cytokine receptor interaction”. So far genes involved in synaptogenesis and calcium channels have been consistently described in the literature^51^,^52^. Some of the chromosome regions overrepresented have been described, but only region 11q25 were in the top regions on ADHDgene database suggesting that our network analysis could be contributing with new candidate genes related to ADHD. More interesting in the network analyses, is the topological properties of ADHD genes. Cai and colleagues^41^ revealed that genes related to diseases tend to be brokers, and this property keeps robust even with the literature bias present in the interactome. Further studies revealed that gene properties in a network are different in autosomal or complex disorders and also that different kinds of genetic variation are related to specific topological areas of the network^22^. It is interesting to observe that the nodes related to ADHD SNVs or CNVs are not central or highly connected as expected for a complex disease. Although they are linked to such nodes, they are located in the borders of the network. This fact supports that the impact of such variants, separately, is not severe. In contrast, it is necessary a combination of factors to trigger the development of the disease. Together with their interactions, the genes with SNVs or in CNVs make complexes related to important biological functions.

Promising results came up when only genes recurrent in at least two different analyses and their direct interactions on PPI databases have been used, since the connections in the neighborhood of these genes could show pathways with more confidence. Some of our seed genes have already been associated with other neurodevelopmental disorders such as schizophrenia and autism. It is important to note that *Cytoplasmic FMR1-interacting protein 2 (CYFIP2)* was identified as an interaction of *FMRP*, clearly related to Autism. Importantly, CYFIP2 is a component of WAVE regulatory complex, a key regulator of actin cytoskeleton. Han and colleagues^53^ showed that CYFIP2 could be implicated in the dendritic spine regulation in cortical neurons and suggest that misregulation of CYFIP2 function and its mGluR-induced expression contribute to the neurobehavioral phenotypes of FXS. OBSL1 is important for the regulation of a signaling mechanism that orchestrates the morphogenesis of the Golgi apparatus and pattern of dendrites^54^. Interestingly *NPSR1* and *UQCRC2* seem to be more specific for ADHD. NPSR1 modulated by calcium signaling pathways has been involved in Modulation of prefrontal functioning in attention systems and motor impairments^55^ and UQCRC2 has been related to methylphenidate treatment^56^.

Yang and colleagues^14^ also combined CNVs and SNPs to study ADHD. Their study, which also used networks to investigate the role of different types of variants, reported synapse (15 genes) and neuron projection (16 genes) as GO (Cellular component) terms associated with regulatory regions. Interestingly, although we confirmed these categories, the overlap of genes is very low – only gene *CNTN2* is in both analyses. Analyzing the enriched pathways, we found genes significantly related to “glutamatergic synapse”, as well as “Serotonergic synapse”. Elia and colleagues^1^ reported networks with genes related to glutamatergic neurotransmission that were affected by CNVs in multiple cohorts. These findings were replicated by Akutagava-Martins and colleagues^57^ in Brazilian samples, which supports the role of glutamate in ADHD. Regarding the glutamatergic pathway genes, our approach also discovered genes not reported by these previous studies in ADHD: PLCB1, PLCB3, GNAI2, GNAI3 and CACNA1A.

In addition to the small sample size, as a limitation of our study, admixed population such as the one from Brazil brings some particularities to the analyses^58^. In this scenario we used as much data integration as possible. Albeit our results confirm the disruption of pathways already associated to ADHD, it also confirms the complexity and heterogeneity of the disease, showing that in different patients, families or populations, disruption in different genes can be conducing to the development of the disease.

In conclusion, using an innovative data integration using public data and the first exome analysis performed in sporadic ADHD trios, we found other genes related to biological pathways previously reported in literature. Although our results confirmed findings published by other research groups, further analyses are essential to confirm the hypotheses and biological functions in larger and different populations. The analyses made exclusively with exome data showed a balance in inherited and *de novo* events, separating in three groups probands without any *de novo* variant, probands with *de novo* CNVs and those who had *de novo* SNVs, and also from children only with inherited variants. This could confirm the role of such variations in ADHD, but further work is necessary to find answers in non-coding regions/regulatory elements in the human genome, as well as the genes in the PPI network affect the processes involved in the disease.

## Additional Information

**How to cite this article**: de Araújo Lima, L. *et al*. An integrative approach to investigate the respective roles of single-nucleotide variants and copy-number variants in Attention-Deficit/Hyperactivity Disorder. *Sci. Rep.* **6**, 22851; doi: 10.1038/srep22851 (2016).

## Supplementary Material

Supplementary Tables 1 and 4

Supplementary Table 2

Supplementary Table 3

Supplementary Table 5

## Figures and Tables

**Table 1:** The 26 *de novo* single-nucleotide variants found in the trios exome sequencing data.

chr	pos	rsID	band	gene	impact	family	frequency
1	2494330	rs2234167	1p36.32	TNFRSF14	NON SYNONYMOUS CODING	NN2624	common
1	41296828	rs34287852	1p34.2	KCNQ4	NON SYNONYMOUS CODING	NN2901	common
2	233537125	rs11550699	2q37.1	EFHD1	NON SYNONYMOUS CODING	NN3752	common
3	193380726	rs200412464	3q29	OPA1	NON SYNONYMOUS CODING	NN3506	very rare
4	169195114	.	4q32.3	DDX60	NON SYNONYMOUS CODING	NN3506	novel
5	825280	rs1809008	5p15.33	ZDHHC11	NON SYNONYMOUS CODING	NN1075	common
7	12409263	rs17165906	7p21.3	VWDE	NON SYNONYMOUS CODING	NN2624	common
7	12409327	rs17165910	7p21.3	VWDE	NON SYNONYMOUS CODING	NN2624	common
7	73969541	rs2301895	7q11.23	GTF2IRD1	NON SYNONYMOUS CODING	1659F	common
9	87338590	.	9q21.33	NTRK2	NON SYNONYMOUS CODING	NN3506	novel
9	101831995	rs10519	9q22.33	COL15A1	NON SYNONYMOUS CODING	1013F	common
9	133577672	.	9q34.12	EXOSC2	NON SYNONYMOUS CODING	NN3506	novel
9	139694569	rs7859194	9q34.3	KIAA1984	NON SYNONYMOUS CODING	1350F	common
13	52544805	rs1801244	13q14.3	ATP7B	NON SYNONYMOUS CODING	NN2624	common
14	20249176	rs2815960	14q11.2	OR4M1	NON SYNONYMOUS CODING	1802F	common
14	24629768	.	14q12	RNF31	NON SYNONYMOUS CODING	1013F	novel
14	25043951	rs61737120	14q12	CTSG	NON SYNONYMOUS CODING	691F	rare
14	102391577	rs3742424	14q32.31	PPP2R5C	NON SYNONYMOUS CODING	1732F	common
15	90818469	rs12595409	15q26.1	NGRN (dist:3026) DQ582071 (dist:18035)	STOP GAINED	NN3506	common
17	7329310	.	17p13.1	C17orf74	SPLICE SITE ACCEPTOR	1372F	novel
17	11784614	.	17p12	DNAH9	NON SYNONYMOUS CODING	1659F	novel
18	61323012	rs3180227	18q21.33	SERPINB3	NON SYNONYMOUS CODING	894F	common
18	61570529	rs6104	18q21.33	SERPINB2	NON SYNONYMOUS CODING	NN3506	common
19	5691424	.	19p13.3	RPL36	NON SYNONYMOUS CODING	NN3538	novel
19	8191184	rs35025963	19p13.2	FBN3	NON SYNONYMOUS CODING	NN1075	common
19	17660300	rs11666267	19p13.11	FAM129C	NON SYNONYMOUS CODING	1659F	common

**Table 2:** Genes with hits in at least two categories of variants found from Brazilian samples exome and from public data.

Gene	public CNVs	public SNPs	exome CNVs	exome SNVs	band	reference
ACOXL (acyl-CoA oxidase-like)	no	no	no	sv2c	2q13	59
ACSM1 (acyl-CoA synthetase medium-chain family member 1)	no	no	no	2vsc	16p12.3	60
ANKRD42 (ankyrin repeat domain 42)	no	no	no	sv2c	11q14.1	
CNGB3 (cyclic nucleotide gated channel beta 3)	yes	no	no	yes	8q21.3	
CYFIP2 (cytoplasmic FMR1 interacting protein 2)	no	no	no	sv2c	5q33.3	61
CYP19A1 (cytochrome P450, family 19, subfamily A, polypeptide 1)	yes	no	no	yes	15q21.2	
DDX60 (DEAD (Asp-Glu-Ala-Asp) box polypeptide 60)	yes	no	no	yes	4q32.3	
DNAH3 (dynein, axonemal, heavy chain 3)	no	no	no	sv2c	16p12.3	
DNHD1 (dynein heavy chain domain 1)	yes	no	no	yes	11p15.4	
EMP2 (epithelial membrane protein 2)	yes	yes	no	no	16p13.13	62
GIMAP6 (GTPase, IMAP family member 6)	no	no	no	2vsc	7q36.1	
GOLGA8DP (golgin A8 family, member D, pseudogene)	yes	no	yes	no	15q11.2	
HEATR1 (HEAT repeat containing 1)	yes	no	no	yes	1q43	
IFLTD1 (lamin tail domain containing 1)	no	no	no	3vsc	12p12.1	
ILDR1 (immunoglobulin-like domain containing receptor 1)	no	no	no	2vsc	3q13.33	
ITGA3 (integrin, alpha 3 (antigen CD49C, alpha 3 subunit of VLA-3 receptor))	yes	no	no	yes	17q21.33	
MSR1 (macrophage scavenger receptor 1)	no	no	no	sv2c	8p22	
MUC6 (mucin 6, oligomeric mucus/gel-forming)	no	no	no	2vsc	11p15.5	
NPSR1 (neuropeptide S receptor 1)	no	no	no	sv2c	7p14.3	
OBSL1 (obscurin-like 1)	no	no	no	sv2c	2q35	
OR8K3 (olfactory receptor, family 8, subfamily K, member 3)	yes	no	no	yes	11q12.1	
PPP6R3 (protein phosphatase 6, regulatory subunit 3)	yes	no	no	yes	11q13.2	
RGS12 (regulator of G-protein signaling 12)	no	no	no	2vsc	4p16.3	63
SDK1 (sidekick cell adhesion molecule 1)	yes	no	no	yes	7p22.2	
SMYD3 (SET and MYND domain containing 3)	yes	no	no	yes	1q44	
TRIM22 (tripartite motif containing 22)	yes	no	no	yes	11p15.4	
TRIM48 (tripartite motif containing 48)	no	no	sv2c	no	11q11	
TRIML2 (tripartite motif family-like 2)	yes	no	no	yes	4q35.2	
UQCRC2 (ubiquinol-cytochrome c reductase core protein II)	yes	no	no	yes	16p12.2	56
VWDE (von Willebrand factor D and EGF domains)	no	no	no	2vsc	7p21.3	

The type of analysis is highlighted with a grey background. All genes are protein coding. The only exception is GOLGA8DP, which is a transcribed unprocessed pseudogene, and OR8K3, which is a polymorphic pseudogene. Legend: sv2c: same variant in 2 children; 2vsc: 2 variants in the same child. In the last column we could observe references of association of the gene and other neurodevelopment disorder.

**Table 3:** KEGG pathways significant after Bonferroni Correction using STRING.

GO id	Term	no. of genes	p-value	p-value Bonferroni
4810	Regulation of actin cytoskeleton	11	6.59E–09	1.89E–06
4730	Long–term depression	7	1.42E–08	4.07E–06
4915	Estrogen signaling pathway	8	1.92E–08	5.51E–06
5016	Huntington’s disease	9	2.83E–07	8.11E–05
4724	Glutamatergic synapse	7	1.14E–06	3.28E–04
4540	Gap junction	6	3.46E–06	9.93E–04
4723	Retrograde endocannabinoid signaling	6	8.07E–06	2.32E–03
4720	Long–term potentiation	5	1.49E–05	4.27E–03
4726	Serotonergic synapse	6	1.67E–05	4.81E–03
