# Corrigendum: An integrative approach to investigate the respective roles of single-nucleotide variants and copy-number variants in Attention-Deficit/Hyperactivity Disorder

**DOI:** 10.1038/srep25861

**Published:** 2016-05-24

**Authors:** Leandro de Araújo Lima, Ana Cecília Feio-dos-Santos, Sintia Iole Belangero, Ary Gadelha, Rodrigo Affonseca Bressan, Giovanni Abrahão Salum, Pedro Mario Pan, Tais Silveira Moriyama, Ana Soledade Graeff-Martins, Ana Carina Tamanaha, Pedro Alvarenga, Fernanda Valle Krieger, Bacy Fleitlich-Bilyk, Andrea Parolin Jackowski, Elisa Brietzke, João Ricardo Sato, Guilherme Vanoni Polanczyk, Jair de Jesus Mari, Gisele Gus Manfro, Maria Conceição do Rosário, Eurípedes Constantino Miguel, Renato David Puga, Ana Carolina Tahira, Viviane Neri Souza, Thais Chile, Gisele Rodrigues Gouveia, Sérgio Nery Simões, Xiao Chang, Renata Pellegrino, Lifeng Tian, Joseph T. Glessner, Ronaldo Fumio Hashimoto, Luis Augusto Rohde, Patrick M. A. Sleiman, Hakon Hakonarson, Helena Brentani

Scientific Reports 6: Article number: 2285110.1038/srep22851; published online: 03072016; updated: 05242016

The Acknowledgements section in this Article is incomplete.

“This work was supported by the National Institute of Developmental Psychiatry for Children and Adolescents, a science and technology institute funded by Conselho Nacional de Desenvolvimento Científico e Tecnológico (CNPq; National Council for Scientific and Technological Development; grant number 573974/2008-0) and Fundação de Amparo à Pesquisa do Estado de São Paulo (FAPESP; Research Support Foundation of the State of São Paulo; grant number 2008/57896-8). Leandro de Araújo Lima was supported by CAPES foundation (process 10682/13-9).”

should read:

“This work was supported by the National Institute of Developmental Psychiatry for Children and Adolescents, a science and technology institute funded by Conselho Nacional de Desenvolvimento Científico e Tecnológico (CNPq; National Council for Scientific and Technological Development; grant number 573974/2008-0) and Fundação de Amparo à Pesquisa do Estado de São Paulo (FAPESP; Research Support Foundation of the State of São Paulo; grant numbers 2008/57896-8 and 2015/01587-0). Leandro de Araújo Lima was supported by CAPES foundation (process 10682/13-9).”

