# Supplementary material for: An integrative approach to investigate the respective roles of single-nucleotide variants and copy-number variants in Attention-Deficit/Hyperactivity Disorder
Source: Sci Rep. 2016 Mar 7;6:22851. doi: 10.1038/srep22851 (PMC4780010; doi:10.1038/srep22851)
Supplement: Supplementary Tables 1 and 4 [file srep22851-s1.doc]

**A****n integrative approach to investigate the respective roles of single-nucleotide variants and copy-number variants in Attention-Deficit/Hyperactivity Disorder**

Leandro de Araújo Lima^1,2^, Ana Cecília Feio-dos-Santos^3^, Sintia Iole Belangero^4,6^ ,Ary Gadelha^4,6^, Rodrigo Affonseca Bressan^4,6^ ,Giovanni Abrahão Salum^4,5^, Pedro Mario Pan^4,6^, Tais Silveira Moriyama^3,4,6^, Ana Soledade Graeff-Martins^3,4^, Ana Carina Tamanaha^4,6^, Pedro Alvarenga^3,4^, Fernanda Valle Krieger^3,4^, Bacy Fleitlich-Bilyk^3,4^, Andrea Parolin Jackowski^4,6^, Elisa Brietzke^4,6^, João Ricardo Sato^4,7^, Guilherme Vanoni Polanczyk^3,4^, Jair de Jesus Mari^4,6^, Gisele Gus Manfro^4,5^, Maria Conceição do Rosário^4,6^, Eurípedes Constantino Miguel^3,4^, Renato David Puga^8^, Ana CarolinaTahira^3^, Viviane Neri Souza^3^, Thais Chile^3^, Gisele Rodrigues Gouveia^3^, Sérgio Nery Simões^1,9^, Xiao Chang^2^, Renata Pellegrino^2^, Lifeng Tian^2^, Joseph T. Glessner^2^, Ronaldo Fumio Hashimoto^1,10^, Luis Augusto Rohde^3,4,5^, Patrick M.A. Sleiman^2,11^, Hakon Hakonarson^2,11^, Helena Brentani^1,3,4,*^

1. Inter-institutional Grad Program on Bioinformatics, University of São Paulo, São Paulo, SP, Brazil
2. Center for Applied Genomics, The Children's Hospital of Philadelphia, Philadelphia, PA, USA
3. Department & Institute of Psychiatry, University of São Paulo Medical School, São Paulo, SP, Brazil
4. National Institute of Developmental Psychiatry for Children and Adolescents (INCT-CNPq), São Paulo, SP, Brazil
5. Department of Psychiatry, Hospital de Clínicas de Porto Alegre, Federal University of Rio Grande do Sul, Porto Alegre, RS, Brazil
6. Department of Psychiatry, Federal University of São Paulo, São Paulo, SP, Brazil
7. Mathematics & Statistics Institute, Federal University of ABC, Santo André, SP, Brazil
8. Hospital Israelita Albert Einstein, Clinical Research, São Paulo, SP, Brazil
9. Federal Institute of Espírito Santo, Serra, ES, Brazil
10. Mathematics & Statistics Institute, University of São Paulo, São Paulo, SP, Brazil
11. Department of Pediatrics, The Perelman School of Medicine, University of Pennsylvania Philadelphia, PA, USA

* Corresponding author

Supplementary Tables

Sup. Table 1. All putative CNVs (de novo or inherited) found on the Brazilian trios children

| chr | start | end | sample | cnv | inheritance | cytoband | genes |
| --- | --- | --- | --- | --- | --- | --- | --- |
| 11 | 55032376 | 55038594 | NN3538 | DEL | denovo | 11q11 | TRIM48 |
| 15 | 22708983 | 22710019 | 703F | DUP | denovo | 15q11.2 | GOLGA8DP |
| 17 | 43697712 | 44116052 | 772F | DEL | denovo | 17q21.31 | CRHR1|CRHR1-IT1|KANSL1|MAPT|MAPT-AS1|MAPT-IT1|SPPL2C|STH |
| 1 | 1387426 | 1431581 | 1084F | DEL | father | 1p36.33 | ATAD3B|ATAD3C |
| 1 | 154919893 | 154928567 | 894F | DEL | father | 1q21.3 | PBXIP1 |
| 1 | 155184845 | 155205102 | 894F | DUP | father | 1q22 | GBA|GBAP1 |
| 7 | 72418838 | 72420735 | NN2742 | DUP | father | 7q11.23 | SFTPA2 |
| 10 | 81315609 | 81320163 | 772F | DEL | father | 10q22.3 | TRIM48 |
| 11 | 55032376 | 55038594 | 1848F | DEL | father | 11q11 | CYP2D6 |
| 22 | 42522502 | 42526883 | 772F | DUP | father | 22q13.2 | NSUN5P2|POM121 |
| 19 | 6375713 | 6387570 | 703F | DUP | mother | 19p13.3 | GTF2F1|PSPN |
| 19 | 39225453 | 39229288 | NN2901 | DEL | mother | 19q13.2 | CAPN12 |
| 22 | 21828886 | 21842312 | 1350F | DEL | mother | 22q11.21 | PI4KAP2 |

Sup. Table 4. Counts of inherited variants (CNV and SNV combined), *de novo* SNVs and de novo CNVs in Brazilian children.

|  |  | **GENETIC VARIATION** | | |  | **DAWBA DIAGNOSIS** | | |  |
| --- | --- | --- | --- | --- | --- | --- | --- | --- | --- |
| **Family** | **Gender** | **De novo SNVs** | **De novo CNVs** | **Total inherited** | **Total variants** | **ADHD** | **Emotional disorder** | **Oppositional defiant disorder** | **Comorbidities** |
| NN2624 | M | 4 | 0 | 9 | 13 | 1 | 0 | 0 | 0 |
| 1013F | M | 2 | 0 | 4 | 6 | 1 | 0 | 0 | 0 |
| NN1075 | M | 2 | 0 | 8 | 10 | 1 | 0 | 1 | 1 |
| NN3538 | M | 1 | 1 | 25 | 27 | 1 | 0 | 0 | 0 |
| 1350F | M | 1 | 0 | 6 | 7 | 1 | 0 | 0 | 0 |
| 1732F | M | 1 | 0 | 10 | 11 | 1 | 0 | 0 | 0 |
| NN2901 | F | 1 | 0 | 12 | 13 | 1 | 0 | 1 | 1 |
| NN3752 | F | 1 | 0 | 15 | 16 | 1 | 1 | 0 | 1 |
| 894F | F | 1 | 0 | 40 | 41 | 1 | 0 | 0 | 0 |
| 703F | M | 0 | 1 | 6 | 7 | 1 | 0 | 0 | 0 |
| 772F | F | 0 | 1 | 4 | 5 | 1 | 1 | 0 | 1 |
|  |  | **1.27** | **0.27** | **12.63** | **14.18** | **1** | **0.18** | **0.18** | **0.36** |
| 756F | M | 0 | 0 | 15 | 15 | 1 | 0 | 0 | 0 |
| NN3528 | M | 0 | 0 | 18 | 18 | 1 | 1 | 0 | 1 |
| 543F | M | 0 | 0 | 2 | 2 | 1 | 0 | 1 | 1 |
| NN2742 | M | 0 | 0 | 11 | 11 | 1 | 0 | 1 | 1 |
| 1273F | M | 0 | 0 | 7 | 7 | 1 | 1 | 0 | 1 |
| 698F | M | 0 | 0 | 9 | 9 | 1 | 0 | 1 | 1 |
| 1084F | M | 0 | 0 | 5 | 5 | 1 | 1 | 1 | 2 |
| 1090F | M | 0 | 0 | 9 | 9 | 1 | 0 | 1 | 1 |
| 1848F | F | 0 | 0 | 12 | 12 | 1 | 0 | 0 | 0 |
|  |  | **0** | **0** | **9.77** | **9.77** | **1** | **0.33** | **0.55** | **0.88** |
